# SARS-CoV-2 Nsp1 mediated mRNA degradation requires mRNA interaction with the ribosome

**DOI:** 10.1080/15476286.2023.2231280

**Published:** 2023-07-06

**Authors:** Soraya I. Shehata, Roy Parker

**Affiliations:** a Department of Molecular, Cellular, and Developmental Biology, University of Colorado Boulder, Boulder, CO, USA; Medical Scientist Training Program, University of Colorado Anschutz Medical Campus, Aurora, CO, USA; b Department of Biochemistry, University of Colorado Boulder, Boulder, CO, USA; Howard Hughes Medical Institute, University of Colorado Boulder, Boulder, CO, USA

**Keywords:** SARS-CoV-2, Nsp1, RNA decay, translation, host shutoff, stress granule

## Abstract

Nsp1 is a SARS-CoV-2 host shutoff factor that both represses cellular translation and promotes host RNA decay. However, it is unclear how these two activities are connected and interact with normal translation processes. Here, we performed mutational analyses of Nsp1, and these revealed that both the N and C terminal domains of Nsp1 are important for translational repression. Furthermore, we demonstrate that specific residues in the N terminal domain are required for cellular RNA degradation but not bulk translation shutoff of host mRNAs, thereby separating RNA degradation from translation repression. We also present evidence that Nsp1 mediated RNA degradation requires engagement of the ribosome with mRNA. First, we observe that cytosolic lncRNAs, which are not translated, escape Nsp1 mediated degradation. Second, inhibition of translation elongation with emetine does not prevent Nsp1 mediated degradation, while blocking translation initiation before 48S ribosome loading reduces mRNA degradation. Taken together, we suggest that Nsp1 represses translation and promotes mRNA degradation only after ribosome engagement with the mRNA. This raises the possibility that Nsp1 may trigger RNA degradation through pathways that recognize stalled ribosomes.

## Introduction

SARS-CoV-2 (SARS2) is the origin of the COVID-19 pandemic, which has killed nearly 7 million people globally to date [1]. SARS-CoV-2 is the third β-coronavirus (βCoV) in two decades to cause a respiratory disease epidemic and the first to progress to a global pandemic, and it is unlikely to be the last novel pathogenic βCoV to pose a significant threat to public health [2]. COVID-19 is also expected to progress to endemicity [3]. Therefore, it is critical to understand common virulence factors of coronaviruses with the goal to developing therapies that could be used not only against SARS2 infection, but potentially against other coronavirus infections as well.

Many βCoVs restrict host cell gene expression (called host shutoff) in order to promote viral gene expression, free up cellular translation machinery, and slow the interferon response [4–7]. In both SARS and SARS2, host shutoff is largely mediated by nonstructural protein 1 (Nsp1). Nsp1 is a 20kD protein translated as part of the ORF1a/b polyprotein and is one of the first proteins to be expressed after viral entry into the cell [8,9]. It has three domains: an alpha helical C-terminal domain (CTD), a flexible linker, and a globular N-terminal domain (NTD). Nsp1 is highly conserved between SARS and SARS2 and much of our knowledge of Nsp1 derives from work studying SARS.

Nsp1 restricts host gene expression in two ways: by arresting host translation and by causing the degradation of host RNAs [4,10–14]. The CTD of Nsp1 binds the 40S ribosome and blocks the mRNA entry channel, which is thought to prevent the proper interaction of the 40S ribosome with the mRNA [15–17]. Mutations in the CTD disrupt Nsp1-40S binding and restore cellular translation [13,16]. The NTD has been implicated in mediating viral mRNA escape from translation repression through interactions with the first stem loop in the viral 5’ UTR [18–21].

How Nsp1 expression confers rapid mRNA degradation is still unknown. It has not been shown to have ribonuclease activity in vitro and bears no similarity to any known RNases [22,23]. Knockdown of the 5’-3’ exonuclease Xrn1 only partially blocks Nsp1-induced mRNA degradation for SARS, which suggests that it is not the primary nuclease responsible for mRNA decay [24]. The link between translation repression and mRNA decay is also not well understood. mRNA decay appears to be a separate function of Nsp1 that requires translation repression, but the Nsp1-triggered nuclease that degrades the mRNAs has not been identified [11,12,19,25].

Here, we developed a system for single-cell analyses of Nsp1-mediated translation repression and endogenous RNA decay. In contrast to analyses using transfected reporter mRNAs, this approach allows the examination of host mRNAs in individual cells expressing Nsp1. We used this approach to study the relative contributions of the *N*- and C- terminal domains on translation repression and RNA decay. We found that both the NTD and CTD are necessary for translation repression and that RNA degradation is a distinct function of Nsp1, which can be separated from translation repression by specific mutations. Moreover, we present several observations that Nsp1 mediated RNA degradation requires mRNAs to be engaged with a ribosome. Specifically, we observe that cytosolic lncRNAs, which are not translated, escape Nsp1 mediated degradation. In addition, compounds blocking ribosome attachment to mRNAs limit Nsp1 mediated degradation, while blocking ribosomes during translation elongation still allows mRNA degradation by Nsp1. These findings suggest that Nsp1 represses translation and promotes mRNA degradation only after ribosome engagement with the mRNA. This raises the possibility that Nsp1 may trigger RNA degradation pathways that recognize stalled ribosomes.

## Results

### Nsp1 degrades mRNAs and represses bulk translation

Previous studies showed that Nsp1 encoded by SARS-CoV-1 (SARS) or SARS-CoV-2 (SARS2) inhibits translation and promotes mRNA degradation [10,12,14,16,17]. However, these findings largely relied on reporter mRNAs to assess the extent of translation repression and RNA degradation. Since these analyses typically examine a pool of transfected cells and show a partial effect on translation repression and mRNA degradation, we hypothesized that the effects might be larger in individual cells based on our observations that Nsp1 is sufficient to essentially completely degrade the host *GAPDH* mRNA [4]. Given this, we analysed Nsp1-mediated effects on translation and host mRNA stability on endogenous host mRNAs in cells by examining individual cells transfected with Nsp1 constructs.

**Figure 1.**
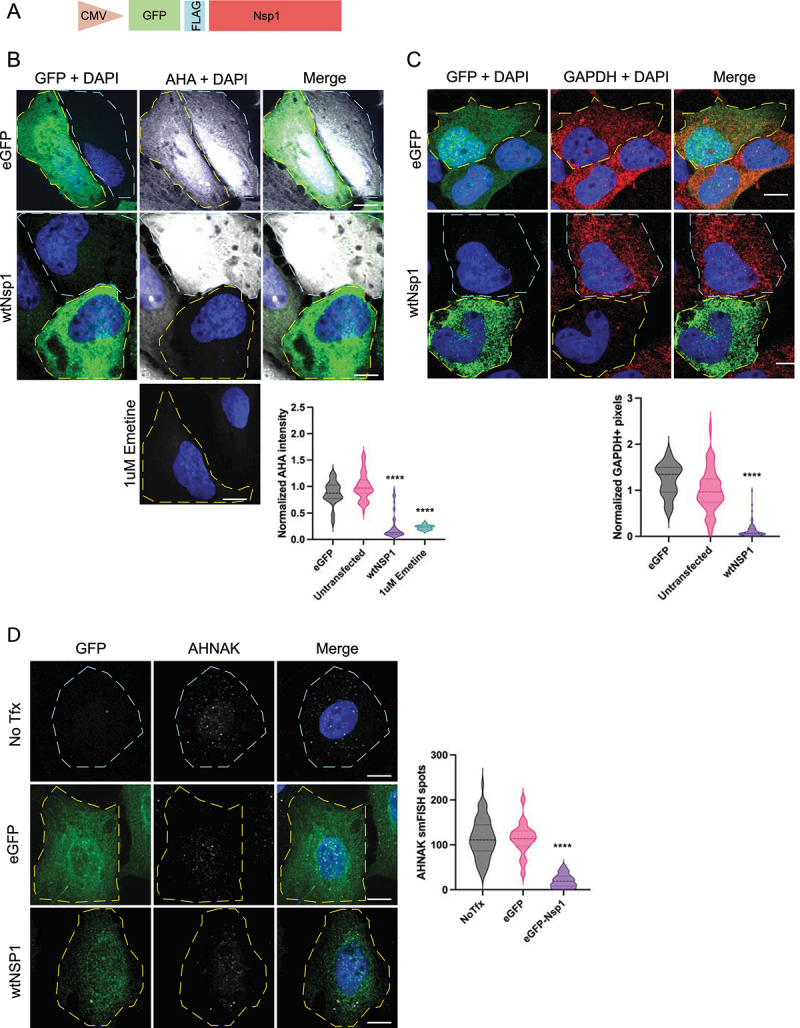
Nsp1 represses endogenous protein translation and causes the degradation of cytosolic mRnas. A) Schematic of GFP-Nsp1 expression construct. B) IF for GFP and AHA-AF647 labelling of Nsp1+, untransfected cells, and cells treated with 1 μM emetine for 90 minutes. Quantified below. C) if for GFP and smFISH for GAPDH, quantified below. D) if for GFP and smFISH for AHNAK, quantified to the right. Ordinary one-way ANOVA compared to eGFP transfection control. ****p < 0.0001; ns = not significant.

For these experiments, we transfected U-2 OS cells with a GFP-Nsp1 expression vector (Figure 1A). We then measured bulk translation of individual cells by pulse labelling cells for 4 hours with L-azidohomoalanine (AHA), a methionine analog, and measured AHA incorporation in proteins via fluorescence (Figure 1B) [26]. Cells expressing Nsp1 (Nsp1+) displayed a 75% reduction in AHA signal compared to untransfected controls, indicating that Nsp1 represses bulk translation of cellular mRNAs. The magnitude of translation repression was similar in cells treated with 1μM emetine, a translation inhibitor (Figure 1B). This demonstrates that Nsp1 substantially reduces the translation of essentially all host mRNAs.

We also assessed mRNA degradation via smFISH by examining mRNAs of different length and abundance. Specifically, we examined *GAPDH* mRNA (1kb, ~4k copies/cell) and *AHNAK* mRNA (18kb; ~100 copies/cell) [27]. Consistent with our previous studies, we observed an 88% reduction in *GAPDH* mRNA levels in Nsp1 expressing cells, confirming that Nsp1 reduces bulk cellular mRNA levels (Figure 1C) [4]. We also observed that Nsp1 similarly reduces cytoplasmic *AHNAK* mRNA levels (Figure 1D). We conclude that Nsp1 causes the degradation of cytoplasmic mRNAs in general, regardless of their abundance or length.

Interestingly, neither *AHNAK* nor *GAPDH* mRNAs notably accumulated in the nucleus, despite the mRNA export block that can arise following bulk cytosolic mRNA degradation (Fig S1B) [28–30]. We did observe that Nsp1 expressing cells had increased nuclear poly-A binding protein (PABP) signal and more nuclear oligo-dT smFISH signal (Figs S1A, S4). This suggests that many, but not all poly-A RNAs, are trapped in the nucleus and that the nuclear export block applies to specific transcripts.

### Montelukast does not alter *GAPDH* mRNA degradation

**Figure 2.**
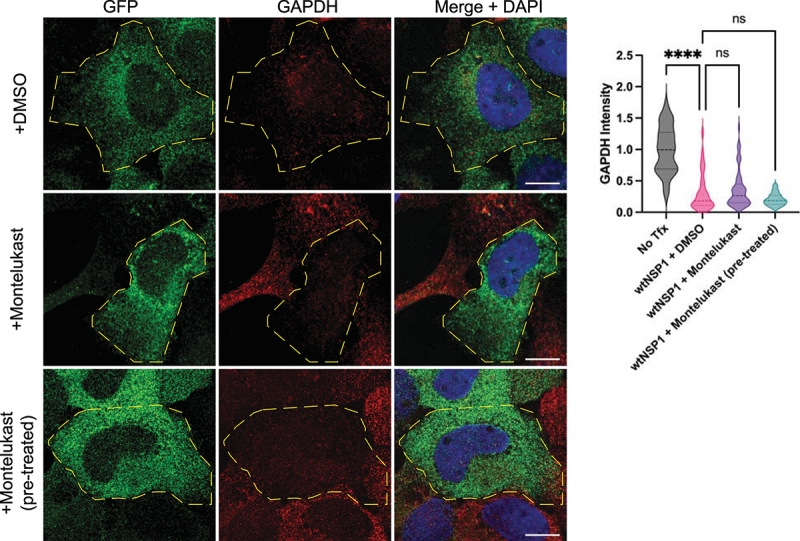
Montelukast treatment does not block GAPDH degradation in Nsp1+ cells. smFISH for GAPDH in cells treated with DMSO or 10 μM montelukast, either before (pre-treated) or after Nsp1 expression, quantified on the right. Ordinary one-way ANOVA compared to wtNsp1 + DMSO.; ****p < 0.0001; ns = not significant.

A recent report suggested that montelukast, a common asthma medication, binds Nsp1 and can rescue translation repression of luciferase in transient transfection experiments [31]. An inhibitor of Nsp1 would be a useful tool for SARS2 research and potentially in the clinic as well, so we examined if montelukast altered mRNA degradation. For this experiment, we treated cells both before and after Nsp1 expression, then assayed *GAPDH* mRNA levels. We observed no significant difference in *GAPDH* degradation in either experiment: mRNA was reduced by the same levels as in Nsp1 controls (Figure 2). Since mRNA degradation appears downstream of translation repression (see below), this suggests that montelukast does not globally inhibit the function of Nsp1. Indeed, we observed that montelukast did not alter Nsp1’s ability to repress translation (Fig S2). We cannot rule out the possibility that montelukast can inhibit the action of Nsp1 on specific luciferase reporter mRNAs.

### Nsp1 does not degrade non-coding RNAs

We examined whether Nsp1 could promote the degradation of untranslated lncRNAs. We examined this possibility given earlier work suggesting that Nsp1 can bind the 40S ribosome to block the mRNA entry channel and prevent ribosome engagement with the mRNA [14–17]. This has suggested two possible models by which Nsp1 represses translation and promotes mRNA degradation. In one model, Nsp1 could prevent the 40S ribosome from interacting with mRNAs and thereby destabilize those mRNAs. This first model predicts that Nsp1 could degrade both translating and untranslating mRNAs. Alternatively, Nsp1 could interact with 40S ribosomes engaged with the mRNA and thereby alter translation in a manner that promotes mRNA degradation. This alternative model predicts that Nsp1 would only be able to degrade translating mRNAs. To test these possibilities, we performed smFISH for the *NORAD* lncRNA (5.3kb; predominantly cytoplasmic, ~ 20 copies/cell), the *GAS5* lncRNA (725bp; cytoplasmic, ~200 copies/cell), and *MALAT1*, a nuclear localized lncRNA (~8kb; predominantly in nuclear speckles, ~90 copies/cell).

**Figure 3.**
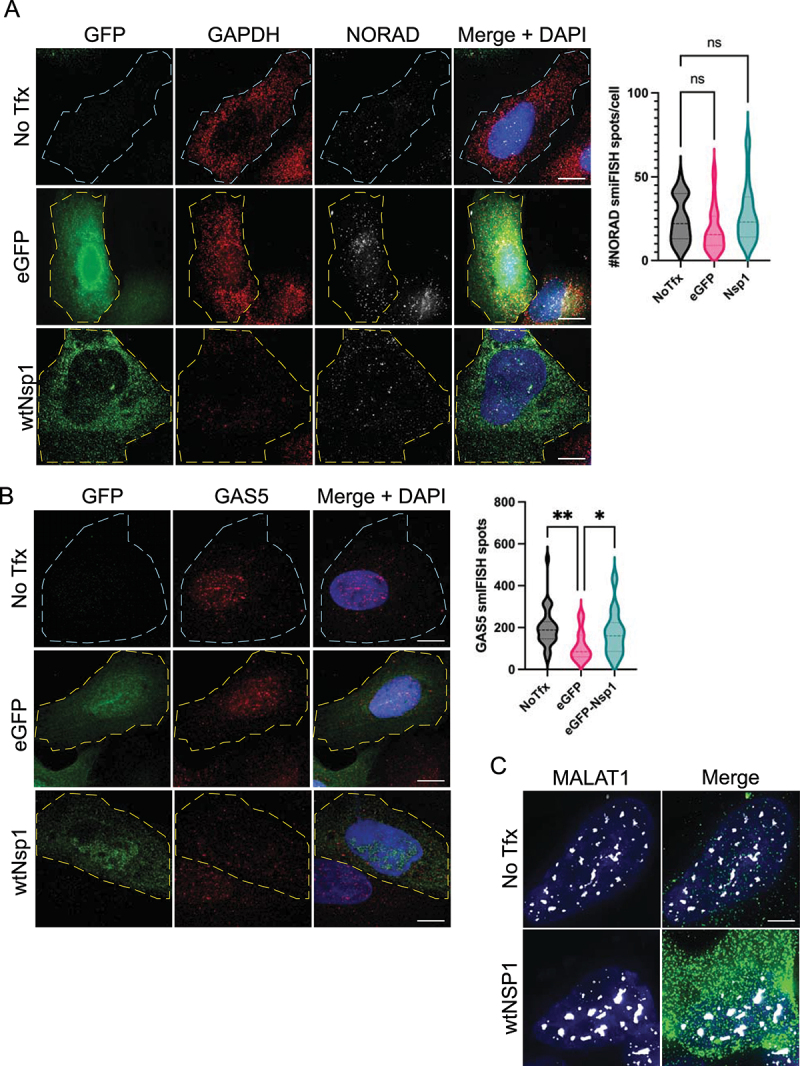
Nsp1 does not degrade lncRNAs. A) smFISH for *GAPDH* and *NORAD*, quantified on the right. B) smiFISH for *GAS5*. C) smFISH for *MALAT1*. Ordinary one-way ANOVA compared to eGFP transfection control. ns = not significant.

We observed that Nsp1 does not reduce any of the untranslating lncRNAs tested (Figure 3). Specifically, Nsp1 expressing cells did not display a difference in the diffuse localization or abundance of *NORAD* or *GAS5* in the cytoplasm in comparison to controls (Figure 3A). Similarly, Nsp1 expressing cells did not display alterations to *MALAT1* RNA abundance or localization (Figure 3B). These data suggest that Nsp1 does not target either cytoplasmic- or nuclear-localized lncRNAs for degradation. We interpret these observations to argue that Nsp1 only degrades translating RNAs in the cytoplasm, which implies that an mRNA needs to associate with a ribosome in a 48S initiation complex, or some alternative configuration, for it to be degraded by Nsp1.

### mRNA degradation and translation repression are two separate functions of Nsp1

**Figure 4.**
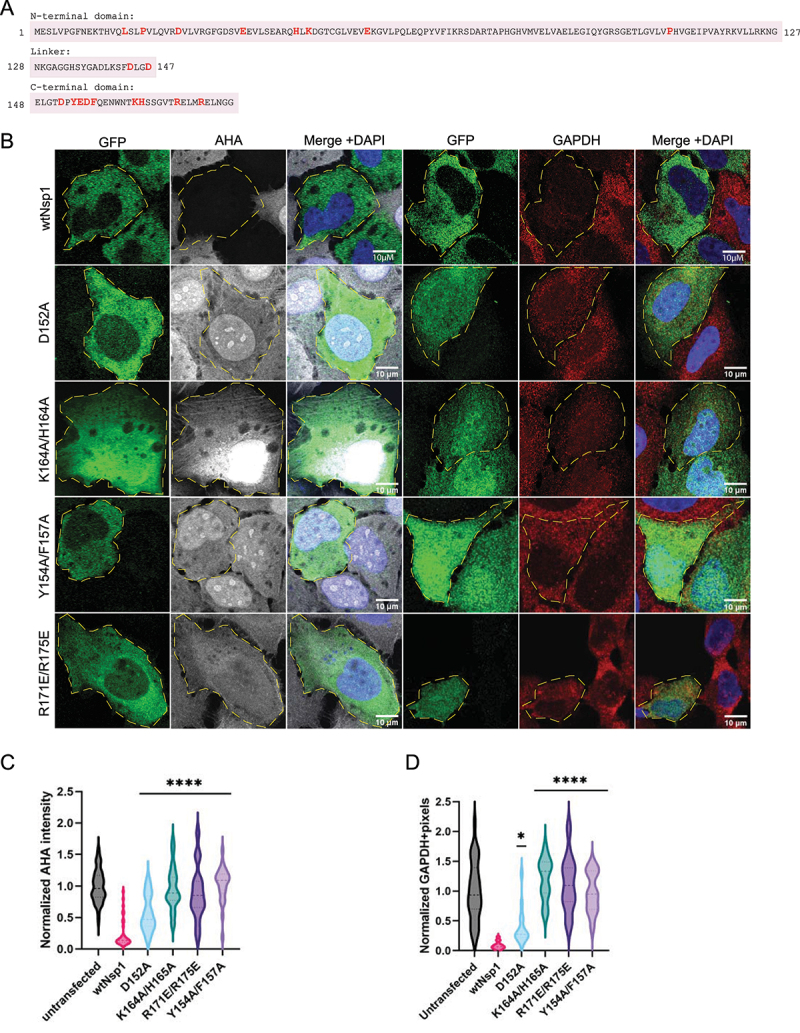
CTD of Nsp1 is critical for translation repression, which is upstream of mRNA decay. A) Amino acid sequence of Nsp1, with mutated residues highlighted in red. The three rows delineate the three domains of Nsp1: the NTD, linker, and CTD, respectively. B) AHA-AF647 labelling of bulk protein synthesis and smFISH for *GAPDH* in cells expressing mutant Nsp1 constructs. C) and D) quantification of AHA labelling and *GAPDH* smFISH. Ordinary one-way ANOVA compared to wtNsp1. *p < 0.05; ****p < 0.0001.

Nsp1‘s sequence and structure are highly conserved throughout SARS1 and SARS2 and have remained very conserved throughout the SARS2 pandemic and evolution of variants [32]. Variants that do express mutated Nsp1 reduce the viral load in infected cells [33]. This suggests that Nsp1 is well evolved as a host shutoff factor and is sensitive to mutations. To test this possibility and to examine the importance of the different domains, we made a series of alanine-scanning mutations in conserved residues. We targeted alleles that are conserved across βCoVs, on the surface of the protein, and charged. We generated 12 mutants across the N-terminal and C-terminal domains of Nsp1 (Figure 4A) and screened each mutant for its ability to repress translation and/or degrade mRNAs by AHA-labelling and *GAPDH* smFISH. We used the K164A/H165A double mutant as a positive control, which is not able to bind the 40S ribosome and therefore fails to inhibit translation or degrade mRNAs [13,16,17].

This analysis revealed three broad categories of Nsp1 mutants: i) mutations that disrupt both translation repression and RNA degradation, ii) mutations that disrupt only mRNA decay, and iii) mutations that have no effect on Nsp1 function. Many of the residues we tested fall in the latter category and have no effect on Nsp1 as a host shutoff factor (Fig S4). We suggest the mutations that do not affect Nsp1’s role in translation repression and/or mRNA degradation are likely to be conserved for other functions of Nsp1.

Consistent with earlier results showing that the CTD of Nsp1 interacts with the ribosome, several mutations in the CTD of Nsp1 abolished both translation repression and mRNA degradation [11,16,17,19]. As previously described, the K164A/H165A double mutant neither repressed translation nor degraded *GAPDH* mRNA (Figure 4). These residues are essential for Nsp1 binding the 40S, and when that interaction is disrupted, Nsp1 expression has no effect on cellular translation or mRNA stability. Additionally, the ribosome-interacting residues at positions Y154, F157, R171, and R175 are also necessary for Nsp1’s ability to both repress translation and trigger RNA degradation [16,17]. Specifically, both Y154A/F157A and R171E/R175E double mutants restored AHA-labelled proteins to levels observed in control cells, and neither showed an ability to degrade *GAPDH* (Figure 4). All six of these mutations are in the distal region of the CTD, suggesting that a 20 amino acid stretch of the CTD must be intact for Nsp1 to suppress translation. Supporting this, the D152A point mutant also reduced the ability of Nsp1 to repress translation and degrade mRNA, although less completely than the more downstream residues (Figure 4B). This suggests that these residues help stabilize the Nsp1-40S interaction but are less critical for binding than the other ribosome-interacting residues, which bind directly to the 40S [17].

**Figure 5.**
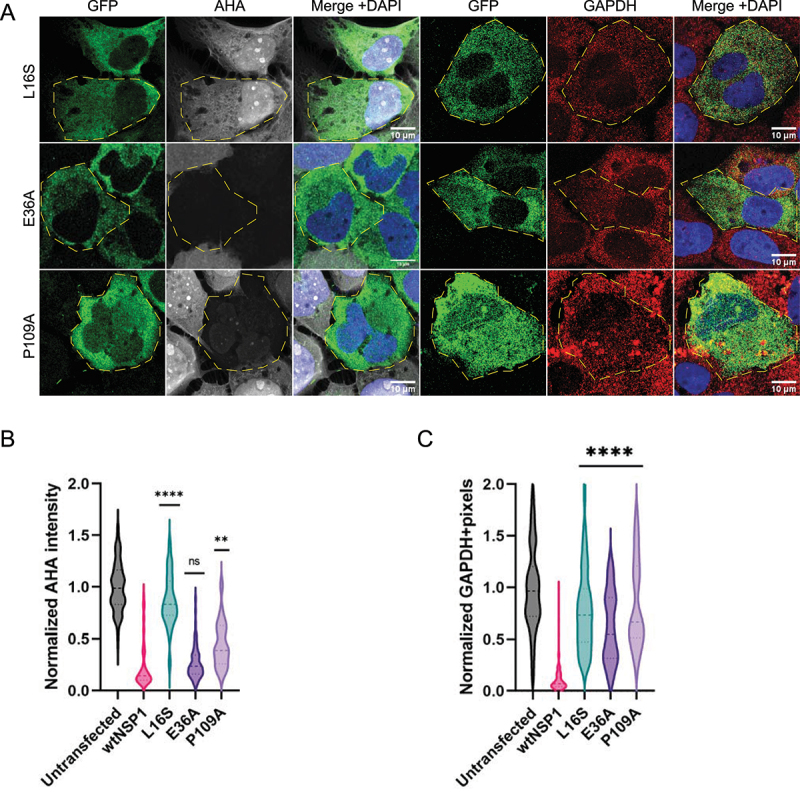
NTD is involved in translation repression and mediates mRNA decay. L16S mutant neither blocks translation repression nor degrades RNA. E36A and P109A mutants abolish *GAPDH* degradation without restoring translation. A) AHA-AF647 labelling of bulk protein synthesis and smFISH for *GAPDH* in cells expressing mutant Nsp1 constructs. B) and C) quantification of AHA labelling and GAPDH smFISH. Ordinary one-way ANOVA compared to wtNsp1. **p < 0.001; ****p < 0.0001; ns = not significant.

A surprising result was that the NTD residue L16 is also essential for Nsp1 translation repression and mRNA decay. We observed that the L16S mutant prevented translation repression by Nsp1 and was not able to degrade RNA (Figure 5). An NTD point mutation that abrogates translation repression has not been observed before. Previously, an R99A mutant was identified and shown to have a moderate effect on restoring translation and mRNA stability [19], but L16S restores both *GAPDH* expression and protein synthesis to normal levels in cells (Figure 5). L16S is expressed similarly to wtNsp1 in cells, suggesting that this point mutation isn’t significantly altering protein expression or folding (Fig S3B). We also tested translation inhibition of nano-luciferase by recombinant L16S in rabbit reticulocyte lysate, and we found that the L16S mutation partially reduces the ability of Nsp1 to repress translation compared to WT protein (Fig S3A). Thus, both in cells and in vitro, the L16S mutation compromises the ability of Nsp1 to repress translation.

We identified two residues, E36 and P109, that are required for Nsp1 mediated RNA decay but not translation shutoff. The Nsp1 mutants E36A and P109A reduced translation to the same extent as wtNsp1 but still had abundant levels of *GAPDH* mRNA. Consistent with these results, the E36A mutant was identified previously as having a mild mRNA degradation defect when combined with a E37A mutation [19]. This demonstrates that Nsp1-mediated translational repression can occur without mRNA decay and that mRNA destabilization is more than a consequence of translation shutoff. We observed two additional residues in the NTD, P19 and D25, that slightly alter mRNA decay but have no effect on translation repression (Fig S4). This is similar to the CTD, where some residues are necessary for the Nsp1-40S interaction and some are needed for stabilization. Therefore, the translation repression and mRNA decay functions of Nsp1 can be decoupled from each other.

### Stalled translation initiation blocks mRNA decay

The above data suggest that Nsp1 has two discrete functions, translation repression and RNA decay. Moreover, since cytosolic lncRNAs are not sensitive to Nsp1, it suggests that an mRNA may need to interact with a ribosome for Nsp1 mediated degradation. To test this possibility further, we blocked translation at specific stages using translation inhibitors to ask how stalling ribosomes in different states affected Nsp1 mediated RNA degradation. In these experiments, we treated Nsp1-expressing cells with a translation inhibitor for five hours to allow for the production of new mRNAs, which would accumulate if Nsp1 mediated degradation was inhibited.

**Figure 6.**
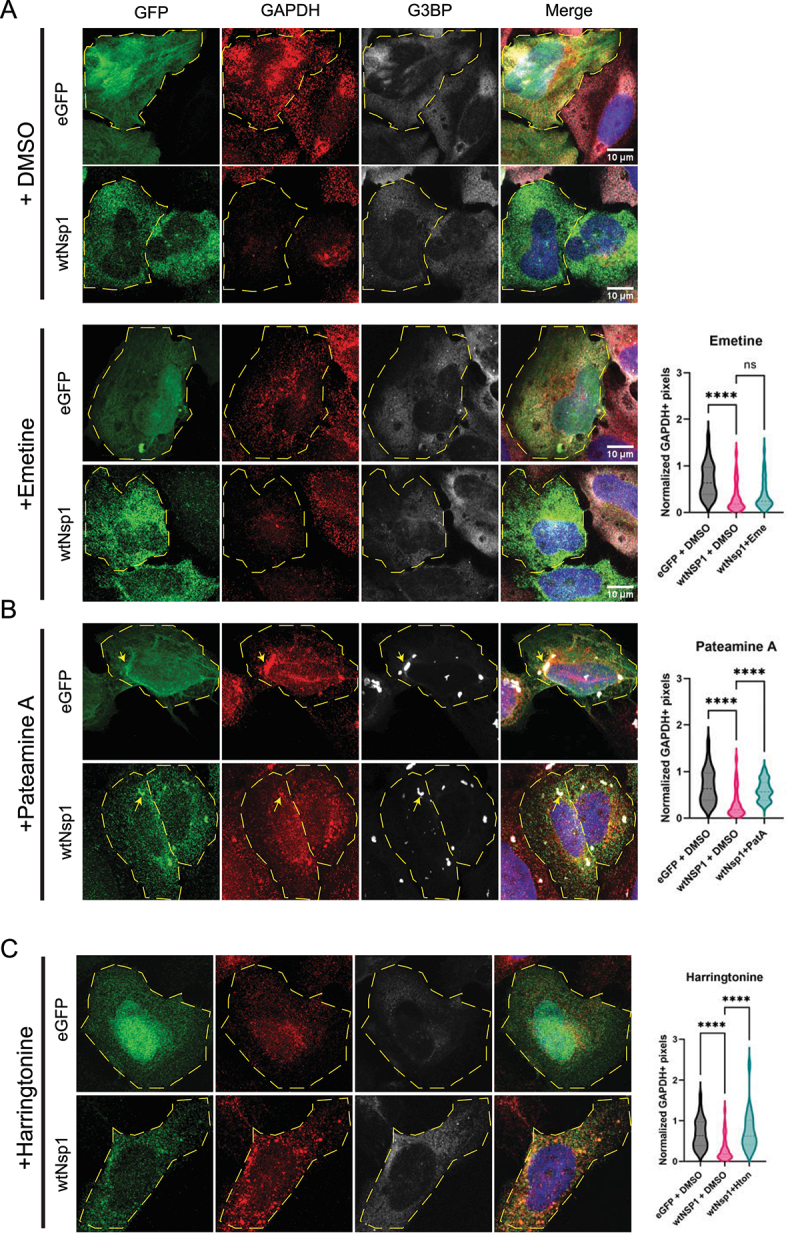
Nsp1 acts downstream of translation initiation and requires a monosome at the start codon before mRNA degradation can occur. smFISH for GAPDH and IF for G3BP in cells expressing GFP-Nsp1 or eGFP and treated with DMSO or 1 μM emetine (A), 100 nM pateamine A (B), or 2 μg/mL harringtonine (C). Quantification on the right. Yellow arrows point to G3BP+ stress granules. Ordinary one-way ANOVA compared to wtNsp1 + DMSO. ****p < 0.0001; ns = not significant.

In the first experiment, we inhibited translation elongation with emetine, which stalls elongating ribosomes along the mRNA by binding the E site and blocking translocation of the tRNA-mRNA [34]. Strikingly, the *GAPDH* mRNA was still strongly reduced in Nsp1 expressing cells treated with emetine (Figure 6A). This suggests that Nsp1 mediated mRNA decay can occur in the presence of stalled ribosomes on the mRNAs.

In a second experiment, we inhibited translation initiation with pateamine A (PatA), which inhibits eIF4A function and blocks recruitment of the 43S pre-initiation complex (PIC) while the ribosomes along the mRNA run off, causing stress granule formation [35]. After five hours of PatA treatment in Nsp1+ cells, we observed increased *GAPDH* mRNAs by smFISH (Figure 6B). We interpret this observation to suggest that at least 48S engagement with the mRNA is required for efficient Nsp1 mediated mRNA degradation. This result implies that Nsp1, bound to the 40S, is included in the PIC and that the PIC must be able to interact with the mRNA before that mRNA is degraded.

Consistent with PatA blocking Nsp1 mediated degradation, we observed that Nsp1 expressing cells treated with PatA form stress granules based on the punctate formation of G3BP1 foci containing *GAPDH* mRNAs (Figure 6B). In contrast, untreated Nsp1 expressing cells are unable to make stress granules, presumably because most of the cellular mRNAs have been degraded [36]. The presence of stress granules validates the increased mRNA levels after PatA treatment in Nsp1 expressing cells. Interestingly, Nsp1 is enriched in stress granules formed in the presence of PatA, suggesting it can stably interact with 48S subunits and/or mRNAs present in stress granules.

### Trapping one monosome at translation initiation site blocks Nsp1-mediated mRNA degradation

The above data indicate that Nsp1-mediated RNA decay occurs between early translation initiation and elongation. To test this further, we treated Nsp1 expressing cells with harringtonine (HTN), which binds the peptidyl transferase centre on the 60S ribosome and inhibits peptide bond formation during the first step of elongation. This traps a single 80S ribosome at the initiation codon while other translating ribosomes run off the mRNA [37]. HTN has been tested as a COVID therapeutic in clinical trials and has been shown to slow viral replication [38,39]. If a 43S PIC interacting with the mRNA is sufficient for Nsp1 mediated degradation, or if mRNA decay occurs before subunit joining, we expect to observe no impact of HTN on RNA degradation. Conversely, if elongating ribosomes are important for mRNA degradation, then we would anticipate HTN would reduce Nsp1 mediated RNA degradation.

We observed that HTN inhibited the Nsp1 dependent degradation of *GAPDH* mRNAs (Figure 6C). Moreover, HTN treatment prevents the increased poly(A)+ RNA accumulation in the nucleus seen with Nsp1 expression (Fig S5). This suggests that a single 80S ribosome stalled at the AUG is not sufficient for Nsp1 mediated RNA degradation and that subunit joining can occur in the presence of Nsp1. Alternatively, it is possible that HTN blocks subunit joining when Nsp1 is bound to the 40S, and that mRNA decay requires at least a fully assembled monosome at the start codon. This result suggests that HTN treatment traps Nsp1 in an assembly with mRNAs that reduces their degradation, and further experiments are needed to characterize this assembly.

## Discussion

Our results strengthen the previous conclusion that the Nsp1 C terminal domain is required for translation repression, which has been established by showing Nsp1 directly interacts with the ribosome and mutations altering that interaction are defective in translation repression [13,16,17]. Here we expand upon that observation, validating that there are at least four other residues, Y154, F157, R171, R175, and to a lesser effect D152, in the C terminal domain that are also required for the full degree of Nsp1 translation repression (Figure 4) and presumably affect the Nsp1-40S interaction [17]. This is consistent with a model wherein additional parts of the Nsp1 CTD are involved in the Nsp1-40S complex [19] and that SARS-CoV-2 variants with CTD deletions replicate less effectively [33].

We also observe that the NTD contains residues important for translation repression. Specifically, we identify the L16S point mutation that diminishes the ability of Nsp1 to repress translation in cells (Figure 5). Moreover, this mutant protein is also partially defective at translation repression in vitro (Fig S3). This is consistent with deletion analysis where removal of the entire Nsp1 N terminal domain leads to reduced translation repression [19]. This suggests a possible interaction between the NTD and CTD that could alter the Nsp1-40S interaction, or the N terminal domain could function in translation repression through an additional, yet to be identified interaction.

Several observations now suggest that RNA degradation is downstream of translation repression and requires additional functions/interactions of Nsp1 after binding the 40S ribosome. This has been initially suggested by the observation that mutations in Nsp1 blocking translation repression, all lead to stabilization of the mRNA (Figures 3, 4) [16,17,19]. Moreover, a key observation is that two specific mutations in Nsp1, E36A and P109A, both still strongly repress host translation but do not degrade host mRNAs (Figure 5). This is consistent with previous work showing that R99 and R124/K125 residues are important for mRNA stability [12,19], revealing that one role of the NTD is to promote mRNA decay. However, the previously described mutations also rescue translation repression, presumably by altering the Nsp1-40S complex. It remains unknown how the NTD residues E36 and P109 contribute to mRNA decay, but one potential mechanism is that they recruit or stabilize a host nuclease. Past experiments have proposed that Xrn1 mediates 5’-3’ mRNA degradation after SARS1 Nsp1 expression [24], and an Nsp1 interactome indicated that Nsp1 interacts with Xrn1 as well as DIS3, part of the 3’-5’ exosome [18]. However, these relationships have not been tested directly in cells, and the mechanism of Nsp1 mediated RNA degradation is still unknown.

Several observations now argue that Nsp1 requires the mRNA to interact with ribosomes to be degraded. First, although Nsp1 RNA decay targets multiple mRNAs, we observed that cytosolic untranslated lncRNAs are not degraded by Nsp1 (Figure 3). Second, RNA-seq data has demonstrated a correlation between translation efficiency and Nsp1-dependent mRNA degradation [40]. Increased translation efficiency indicates more mRNA-ribosome engagement, which is consistent with the model in which Nsp1 mRNA degradation depends upon at least an mRNA-40S-Nsp1 complex. Third, we observed that either preventing 40S ribosome interaction with the mRNA by PatA treatment, or inhibiting the transition to elongation by 80S subunits with HTN stabilizes mRNAs from Nsp1-dependent degradation (Figure 6). These results are consistent with a model wherein elongating 80S ribosomes are required for Nsp1 to trigger mRNA degradation.

An unresolved issue is the exact nature and diversity of the Nsp1-ribosome-mRNA complex(es). Nsp1 competes with eIF3J for ribosome binding, preventing formation of a functional 48S complex [14]. The same study showed through structural analyses that Nsp1 and the cricket paralysis virus (CrPV) IRES mRNA can bind the 40S ribosome simultaneously, but that Nsp1 limits the 40S head conformational change that is necessary for scanning [14]. This finding is inconsistent with the single-molecule observations that mRNA and Nsp1 are mutually exclusive in the mRNA entry channel: when one is bound, the other cannot interact [15]. However, previous ribosome toe-printing analysis with SARS1 demonstrated that a 48S complex (mRNA-43S PIC-Nsp1) can form, but that subunit joining is blocked [11].

This presents a contradiction. Solved structures of Nsp1 bound to the ribosome show that Nsp1 can bind to an 80S ribosome, but they also reveal a clear block to the mRNA entry channel suggesting that Nsp1-40S interaction would prevent the 40S ribosome interacting with mRNA [16,17]. Additionally, it is still unclear if Nsp1 permits the formation of a functional 48S, wherein the mRNA is properly accommodated on the 40S ribosome. However, our observations suggest that ribosome engagement with the mRNA is required for Nsp1 to promote mRNA degradation (Figures 3 & 6). Although the basis of this apparent contradiction remains to be solved, we suggest three possibilities. First, it could be that Nsp1 interacts with a second 40S pre-initiation complex and the interaction of the Nsp1-40S complex with an 80S on the mRNA triggers mRNA degradation, which could explain Nsp1-80S interactions [16,17]. Alternatively, Nsp1 may insert into the mRNA entry channel at a later stage of the mRNA decay process, and therefore the structure does not reveal an initial required interaction. Lastly, Nsp1 could force mRNA to bind a different site on the ribosome through interactions with RBPs and eIFs. Understanding the Nsp1-ribosome-mRNA assembly will require further structural analyses.

The requirement for ribosome mRNA engagement for Nsp1 mediated degradation raises the possibility that Nsp1 may alter ribosome dynamics. Interestingly, when ribosome movement is inhibited, the collision of either 80S subunits, or 40S and 80S subunits, can trigger ribosome quality control pathways that lead to mRNA cleavage and degradation [41]. This, and other observations, lead to the possibility that Nsp1 leads to activation of RQC pathways and subsequent mRNA decay. For example, Nsp1 from SARS is known to induce endonucleolytic cleavage of mRNAs in specific contexts [11,12,23]. Moreover, Nsp1 overexpression has been proposed to resolve stalled ribosome complexes [42]. Further experiments are needed to determine if Nsp1’s involvement with quality control factors is what causes mRNA cleavage and degradation.

## Materials and methods

### Cell culture and transfections

U2OS cell lines (ATCC) were maintained at 5% CO_2_ and 37°C in Dulbecco’s modified Eagle’s medium (DMEM) supplemented with 10% v/v foetal bovine serum (FBS) and 1% v/v penicillin/streptomycin. Cells were routinely tested for mycoplasma contamination by our core facility. Cells were transfected with X-tremeGENE HP transfection reagent (2 μl per 1 μg DNA) (Sigma 6366244001). Four hours after adding transfection mix, the media was replaced. Cells were fixed for downstream analyses 24 hours after transfection.

### Cloning and mutagenesis

Full-length wtNsp1, GFP, and eGFP were synthesized by Integrated DNA Technologies (IDT) as g-Block gene fragments. FLAG-Nsp1 was cloned into the pcDNA3.1+ vector as described in Burke, et al 2021. GFP and eGFP were ligated upstream of Nsp1 using EcoRI and XhoI restriction sites. All plasmids sequences were verified using Sanger sequencing or whole plasmid sequencing. Site-directed mutagenesis was used to generate point mutants and double mutants (primers listed in Table S1).

### Fluorescence microscopy sample preparation

Sequential smFISH and IF on U2OS cells were performed as described previously with the following modifications [27]. Cells were grown on glass coverslips in a 24-well plate, then fixed for 10 minutes with 4% paraformaldehyde. *GAPDH* and oligo-dT smFISH probes labelled with Quasar-570 were purchased from Stellaris. Custom *AHNAK* smFISH probes [27] were purchased as DNA oligos (Table S2) from IDT and labelled with Atto-633 using 5-Propargylamino-ddUTP-ATTO-633 using terminal deoxynucleotidyl transferase (Thermo Fisher Scientific) as described previously [43]. Custom *NORAD* and *GAS5* smiFISH probes were purchased as DNA oligos (Table S2) and labelled with ATTO-647n as described previously [44].

After smFISH/smiFISH was performed, coverslips were rinsed with phosphate buffered saline (PBS) before being fixed again for 10 minutes in 4% paraformaldehyde. Cells were washed twice with PBS then incubated for one hour at room temperature with primary antibody, washed three more times with PBS then incubated with secondary antibody for 1 hour. After washing 3× with PBS, coverslips were mounted on slides with ProLong Glass Antifade Mountant (ThermoFisher P36982).

Antibodies used: polyclonal anti-GFP (rabbit, 1:500, Invitrogen A-11122); monoclonal anti-GFP (mouse, 1:500, Santa Cruz sc-9996); monoclonal anti-G3BP (mouse, 1:500, Abcam ab56574); polyclonal anti-PABP (rabbit, 1:500, Abcam ab21060). Secondary antibodies were all used at 1:1000 dilution. Goat anti-rabbit IgG Alexa Fluor 488 (Invitrogen A11008); goat anti-mouse IgG Alexa Fluor 488 (Invitrogen A1101); Goat Anti-Mouse IgG H&L Alexa Fluor 647 (Abcam ab150115); Goat Anti-Rabbit IgG H&L Alexa Fluor 647 (Abcam ab150079).

### L-azidohomoalanine (AHA) labelling

AHA labelling was performed as described by the manufacturer (Fisher C10102). Briefly, cells growing on glass coverslips were incubated in methionine-free DMEM (Gibco 21013024) with 10% v/v FBS for one hour. 50 μM Click-iT AHA (Invitrogen C10102) was added to cells for four hours before fixation with 4% paraformaldehyde. Cells were permeabilized with 0.1% Triton-X 100 in PBS for 15 minutes, then washed with 3% BSA in PBS before adding the Click-iT Cell Reaction cocktail (Invitrogen C10269). Click reaction with Alexa-Fluor 647 alkyne (Invitrogen A10278) was performed as described by the manufacturer (https://assets.thermofisher.com/TFS-Assets%2FLSG%2Fmanuals%2Fmp10269.pdf).

### Drug treatments

U2OS cells were transiently transfected with GFP-Nsp1 as described above. After 20 hours, media was replaced with media containing either 1:1000 DMSO, 1 μM emetine (Cayman Chemical Company 316-42-7), 100 nM pateamine A, or 2 μg/mL harringtonine (Abcam ab141941). Cells were incubated at 37°C for 5 hours before fixation. For montelukast treatment, cells were treated for 5 hours with 10 μM montelukast (Sigma SML0101) as described above. For the pretreatment condition, cells were seeded onto glass coverslips in 10 μM montelukast 24 hours before Nsp1 transfection.

### Microscopy and image analysis

Fixed cell imaging was performed using a 100× oil objective on a Nikon A1R Laser Scanning Confocal microscope with an Andor iXon 897 Ultra detector or a Nikon SoRa Spinning Disc Confocal microscope with an Andor iXon Life 897 EMCCD Camera. We imaged 10–20 z-slices at a distance of 0.2 μM/slice. All images shown are a max projection in Z of the z-stack.

We used ImageJ (version 2.9.0/1.53t) to quantify *GAPDH* smFISH spots. After creating a max intensity projection in Z, we set an intensity threshold for the experiment and analysed the number of pixels above that threshold within manually defined regions of interest (ROIs). Each ROI represents a single cell. To quantify *AHNAK*, *NORAD* and *GAS5* smFISH spots, we first made a mask of the nuclei using CellProfiler (version 4.2.5) to specifically analyse the cytosol (or nuclei), set an intensity threshold as described above, then used the ImageJ feature Analyse Particles to count the number of smFISH spots above the intensity threshold in each ROI.

AHA intensity was quantified using ROIs manually defined in ImageJ. We then measured the average intensity of each ROI.

### SDS-PAGE and Western blotting

Cells were transfected with GFP-Nsp1 constructs as described above in 6 well plates. After 24 hours, cells were trypsinized and pelleted, then resuspended in 200 μl lysis buffer (1.25% SDS and 4% β-mercaptoethanol in water). Cells were frozen at −80°C to complete lysis then boiled at 95°C for 10 minutes prior to running on a NuPAGE 4–12% Bis-Tris Gel (Fisher Scientific, NP0322BOX) and transfer to a nitrocellulose membrane (ThermoFisher, IB23002). The membrane was blocked with 5% milk in Tris-buffered saline with 0.1% Tween-20 (TBS-T) for 1 hour at room temperature then incubated with primary antibody in 5% milk and TBS-T overnight at 4°C. The membrane was washed three times with TBS-T then incubated with secondary antibody for 1 hour at room temperature, then washed again with TBS-T before incubation with the chemiluminescence substrate (Biorad 170506) for 5 minutes at room temperature prior to imaging with an iBright FL1500 Imaging System. Rabbit anti-Nsp1 (GeneTex GTX135612) and GAPDH – HRP antibody (Santa Cruz, sc -47724 HRP), anti-rabbit IgG HRP-linked secondary antibody (Cell Signaling Technology, 7074S) were used at 1:1000.

### Protein purification

Recombinant wtNsp1 and mutants were expressed and purified as described previously [19]. The pGEX-Nsp1 expression plasmid (Addgene 175512) was mutated using the Quikchange site directed mutagenesis kit (Agilent 200153), then transformed in BL21 competent cells (Agilent 230,240). Overnight Express TB (EMD Millipore 71,491–4) cultures were inoculated and grown at 37°C to an OD600 of 0.6, then moved to 18°C for 24 hours. Cells were pelleted, washed with PBS and a protease inhibitor cocktail (Sigma Aldrich 11836170001), then pelleted again. Cells were resuspended in lysis buffer (500 mM NaCl, 5 mM MgCl_2_, 20 mM HEPES, 0.5% Triton-X 100, 5% glycerol, 1 mM TECEP, pH 7.5), then lysed at 4°C for 12 minutes with a macrotip sonicator (3 second pulse and 17 second rest), then centrifuged for 35 minutes at 39,000×g. Glutathione Sepharose (Sigma, GE17075601) beads were washed with 5 column volumes (CV) of lysis buffer on a glass Econo-Column (Biorad 7372512), then incubated with the cleared lysate for 2 hours at 4°C. Flow-through was collected and the beads were washed with 10 CV of lysis buffer, then washed with 20 CV of wash buffer (250 mM NaCl, 5 mM MgCl_2_, 5% glycerol, 20 mM HEPES, 1 mM TECEP, pH 7.5), and finally resuspended in 1 CV of wash buffer with HRV 3C Protease (Millipore 71493). Nsp1 was eluted overnight at 4°C on a nutator then collected. The beads were washed with 1 CV of wash buffer. The two elutions were pooled and concentrated with an Amicon Ultra-4 10K centrifugal filter (Millipore UFC801024). Concentrated protein was then purified by size exclusion column chromatography. Purity and mass of the eluted protein were confirmed by SDS-PAGE gel electrophoresis and SimplyBlue SafeStain (Invitrogen LC6060).

### In vitro transcription

Nanoluciferase (NanoLuc) mRNA was generated with T7 RNA polymerase (ThermoFisher AM1334) as previously described [19]. DNA was PCR amplified from a plasmid with the NanoLuc coding sequence (Addgene 175431), then gel purified before T7 transcription. After transcription, RNA was purified using the MEGAClear Transcription Clean-Up Kit (Thermo Scientific AM1908), then capped and 2’O methylated with the Vaccinia Capping System (NEB M2080S) and the mRNA Cap 2´-O-Methyltransferase (NEB M0366). Capped NanoLuc mRNA was isolated using Direct-zol RNA Miniprep kits (Zymo R2050).

### In vitro translation assay

Nuclease treated rabbit reticulocyte lysate (RRL) (Promega L4960) was used as described by the manufacturer for non-radioactive reactions with the following modifications. RRL lysate was incubated with 640 nM of Nsp1 or glutathione-S transferase (Sigma G6511) for 10 minutes on ice before adding nanoluciferase mRNA. The reaction proceeded for 90 minutes at 30°C before the addition of the NanoGlo luciferase substrate (Promega N1110). Luminescence was visualized with a BMG plate reader.

## Supplementary Material

Supplemental Material

## Data Availability

The data that support the findings of this study can be accessed online at https://doi.org/10.6084/m9.figshare.c.6569116.
